# Synthesis of densely functionalized enantiopure indolizidines by ring-closing metathesis (RCM) of hydroxylamines from carbohydrate-derived nitrones

**DOI:** 10.1186/1860-5397-3-44

**Published:** 2007-12-12

**Authors:** Marco Bonanni, Marco Marradi, Francesca Cardona, Stefano Cicchi, Andrea Goti

**Affiliations:** 1 Dipartimento di Chimica Organica "Ugo Schiff", Laboratorio di Progettazione, Sintesi e Studio di Eterocicli Biologicamente Attivi (HeteroBioLab), Università di Firenze, via della Lastruccia 13, I-50019 Sesto Fiorentino (Firenze), Italy

## Abstract

**Background:**

Indolizidine alkaloids widely occur in nature and display interesting biological activity. This is the reason for which their total synthesis as well as the synthesis of non-natural analogues still attracts the attention of many research groups. To establish new straightforward accesses to these molecules is therefore highly desirable.

**Results:**

The ring closing metathesis (RCM) of enantiopure hydroxylamines bearing suitable unsaturated groups cleanly afforded piperidine derivatives in good yields. Further cyclization and deprotection of the hydroxy groups gave novel highly functionalized indolizidines. The synthesis of a pyrroloazepine analogue is also described.

**Conclusion:**

We have developed a new straightforward methodology for the synthesis of densely functionalized indolizidines and pyrroloazepine analogues in 6 steps and 30–60% overall yields from enantiopure hydroxylamines obtained straightforwardly from carbohydrate-derived nitrones.

## Background

Indolizidine alkaloids have widespread occurrence in nature. They can be found in widely different organisms such as bacteria, fungi, higher plants, invertebrates and vertebrates.[1] For instance, the plant-derived polyhydroxylated indolizidines are well known as potent glycosidases inhibitors, and for this reason they are potential therapeutic agents. [2–4] A great deal of research is still devoted to the structural elucidation of these alkaloids as well as to their total syntheses. [5–18]

**Scheme 1:**
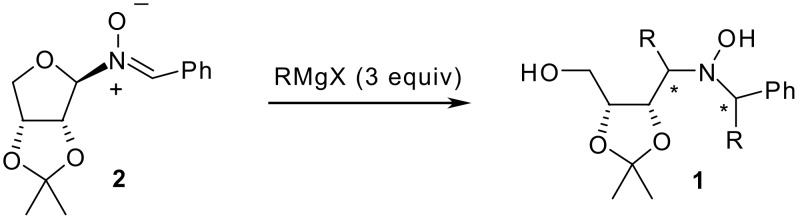
Synthesis of symmetrically α,α'-disubstituted hydroxylamines **1**.

We accomplished the total syntheses of some indolizidine alkaloids and of several non-natural analogues employing chiral nitrones as key intermediates, either as dipolarophiles in 1,3-dipolar cycloaddition chemistry [19–20] or as electrophiles in the addition of organometallic reagents. [21–22] Recently, we developed a general protocol for the synthesis of α,α'-disubstituted enantiopure hydroxylamines **1** through the stereoselective double addition of an excess of a Grignard reagent to *C*-phenyl-*N*-erythrosylnitrone **2** (Scheme 1).[23] With this methodology, several symmetrically α,α'-disubstituted hydroxylamines **1** were afforded.

**Scheme 2:**
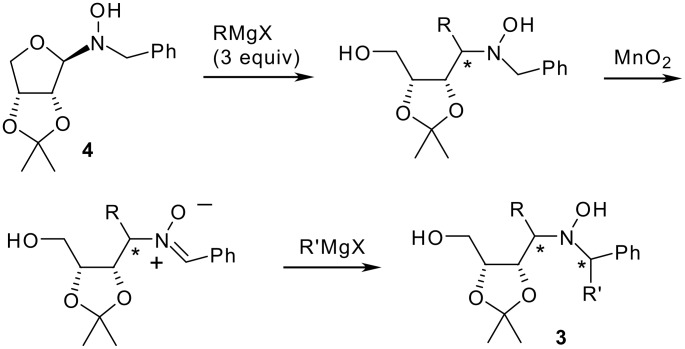
Synthesis of unsymmetrically α,α'-disubstituted hydroxylamines **3**.

An alternative protocol for the synthesis of unsymmetrically α,α'-disubstituted hydroxylamines **3**, resulting from the sequential addition of two different Grignard reagents, was also developed in a stepwise process, based on an addition-oxidation-addition sequence starting from *N*-glycosylhydroxylamine **4** (Scheme 2).[24]

Addition of unsaturated Grignard reagents afforded synthetically useful hydroxylamine intermediates, which may serve as substrates for nitrogen ring forming reactions. We report in this article a straightforward access to indolizidine derivatives and a pyrroloazepine analogue through a key ring closing metathesis (RCM) of sugar derived hydroxylamines **1** and **3** bearing suitable unsaturated substituents at the α and α' positions.

## Results and discussion

**Scheme 3:**
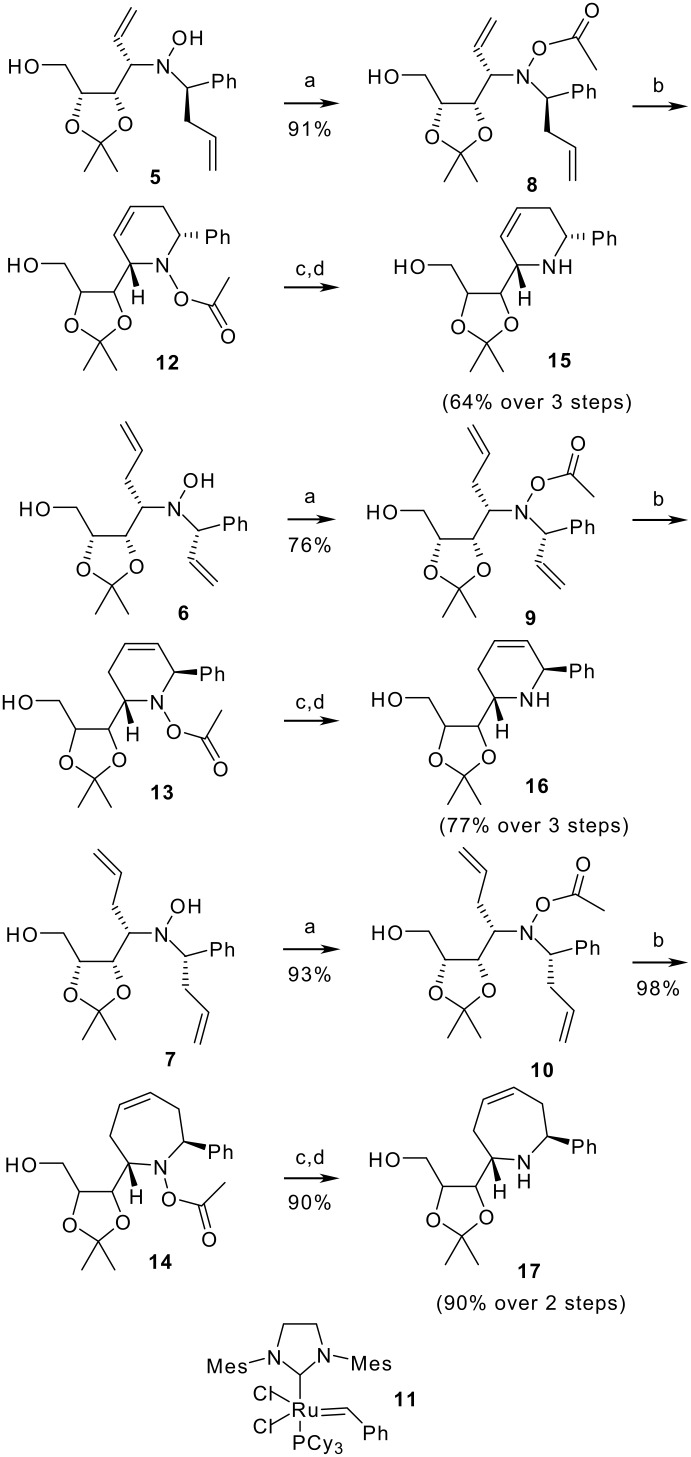
Synthesis of piperidines **15–16** and azepine **17**. Reagents and conditions: a) Ac_2_O, THF, 1 h, rt for **8** and **9**, reflux for **10**; b) 2^nd^ generation Grubbs' catalyst **11** (5 mol%), CH_2_Cl_2_, reflux, 5.5 h; c) KHCO_3_, MeOH, rt, 12 h; d) Zn, AcOH, rt, 2 h.

Unsymetrically α,α'-disubstituted hydroxylamines **5** and **6** (Scheme 3) were synthesized according to our recently reported procedure based on the addition-oxidation-addition sequence starting from *N*-glycosylhydroxylamine **4** (Scheme 2),[24] while hydroxylamine **7** was obtained using an excess of allylmagnesium bromide in the addition to *C*-phenyl-*N*-erythrosylnitrone **2**.[23] It should be noted that the stepwise process furnishes configurationally diversified stereoisomers at the benzylic position (e. g. **5** and **6**), due to a high stereoselectivity in the first addition step but a poor one in the second.[24] Specifically, **5** was isolated as the major isomer from a ca 2:1 diastereomeric mixture, while **6** was obtained from an equimolecular mixture with its diastereoisomer.[24] Assignment of configuration has been secured by comparison with the double adducts of the one-pot process and by careful NMR studies of the final cyclic products after RCM. The scarce stereoselectivity of the second addition in the stepwise process, giving rise to two diastereoisomers, opens the way to the synthesis of diastereomeric indolizidines.

The RCM reaction has been successfully employed for the synthesis of polyfunctional indolizidines. [25–29] In order to accomplish successfully the key RCM reactions, preliminary protection of the hydroxylamine OH group was required. Selective acetylation of hydroxylamines **5–6** was achieved with acetic anhydride in THF at room temperature, while for hydroxylamine **7** it was necessary to heat the mixture at reflux. No acetylation of the primary alcohol was observed under these conditions.

Ring-closing metathesis (RCM) of *O*-acetylhydroxylamines **8–10** using the second generation Grubbs' catalyst **11** [30] in refluxing CH_2_Cl_2_ afforded compounds **12–14** in nearly quantitative yields (Scheme 3). However, compounds **12** and **13** suffered from low stability and for this reason the crude reaction mixtures were directly employed in the following steps.

Identity of compounds **12** and **13** was firmly established after their transformation into the corresponding amines and further elaboration. After deacetylation with KHCO_3_, *in situ* reduction of the N-O bond with zinc dust afforded tetrahydropyridines **15** and **16** (Scheme 3). Analogously, deprotection of **14** and *in situ* reduction with zinc dust gave tetrahydroazepine **17** (Scheme 3).

**Scheme 4:**
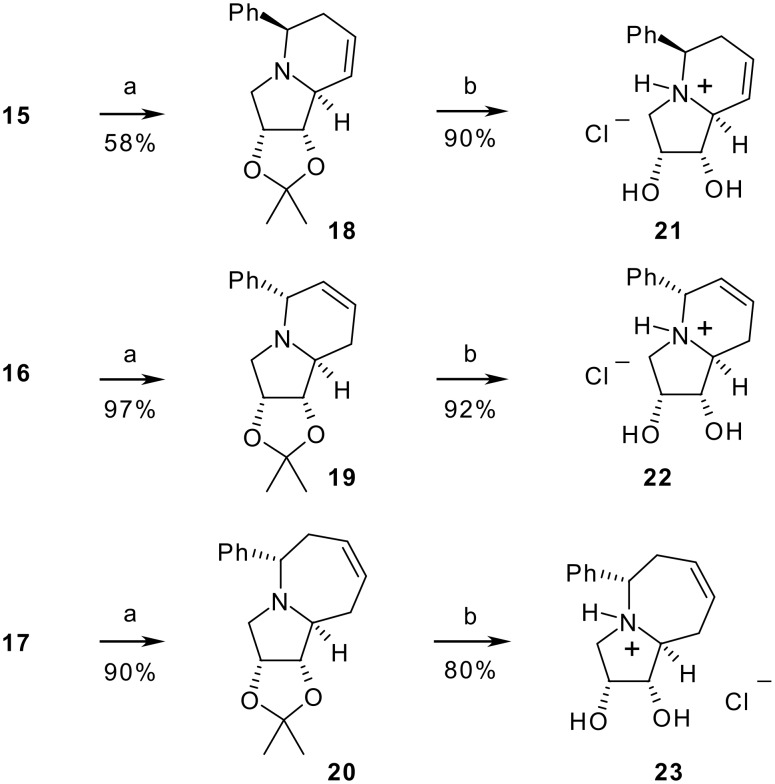
Synthesis of indolizidines **21–22** and pyrroloazepine **23**. Reaction conditions: a) Tf_2_O, Py, rt, 2 h; b) Conc. HCl, MeOH, rt, 2 h.

Cyclization to give the fused 5-membered ring was achieved by treatment of compounds **15–17** with trifluoromethanesulfonic anhydride in pyridine at room temperature (Scheme 4). The structure of protected indolizidines **18–19** and of pyrroloazepine **20** (and therefore of compounds **12–14**) was unambiguously determined by spectral data, including 2D COSY and 1D NOESY experiments (See Experimental).

Final deprotection of **18–20** with an acidic solution of MeOH afforded protonated indolizidines **21–22** in good yields (Scheme 4). Analogously, deprotection of **20** gave pyrrolazepine **23**, which displayed good inhibition of α-glucosidase from yeast (90% at 1 mM).[23] Compounds **17** and **23**, containing an azepane moiety, might be of biological interest as shown recently. [31–35] Indolizidines **21–22** differ in the absolute configuration at C5 and in the position of the double bond, illustrating the structural diversity attainable with this strategy. It should be noted that similar dihydroxyhexahydroindolizines maintained glucosidase inhibition activity in analogy to the completely unsaturated compounds.[22] Moreover, it has been recently proved that dihydroxypyrrolidines bearing aromatic rings have interesting antitumor activities. [36–37] Work is underway to evaluate the biological activity of the newly synthesized compounds. In addition, the presence of a double bond should allow the introduction of additional hydroxy groups or other functionalities by appropriate elaboration.

## Experimental

[See Supporting Information File 1 for full experimental data]

## Supporting Information

File 1 Synthesis of densely functionalized enantiopure indolizidines by ring-closing metathesis (RCM) of hydroxylamines from carbohydrate-derived nitrones. Experimental Sections. Experimental procedures, characterization of new compounds.
