# Supplementary material for: Synthesis of densely functionalized enantiopure indolizidines by ring-closing metathesis (RCM) of hydroxylamines from carbohydrate-derived nitrones
Source: Beilstein J Org Chem. 2007 Dec 12;3:44. doi: 10.1186/1860-5397-3-44 (PMC2151072; doi:10.1186/1860-5397-3-44)
Supplement: File 1 — Synthesis of densely functionalized enantiopure indolizidines by ring-closing metathesis (RCM) of hydroxylamines from carbohydrate-derived nitrones. Experimental Sections. Experimental procedures, characterization of new compounds. [file Beilstein_J_Org_Chem-03-44-s001.pdf]

Synthesis of densely functionalized enantiopure indolizidines by ring-closing metathesis (RCM) of hydroxylamines from carbohydrate-derived nitrones

Marco Bonanni, Marco Marradi, Francesca Cardona, Stefano Cicchi and Andrea Goti*

Dipartimento di Chimica Organica "Ugo Schiff", Laboratorio di Progettazione, Sintesi e Studio di Eterocicli Biologicamente Attivi (HeteroBioLab), Università di Firenze, via della Lastruccia 13, I-50019 Sesto Fiorentino (Firenze), Italy

Email address: andrea.goti@unifi.it

All reactions requiring anhydrous conditions were carried out under nitrogen and solvents were appropriately dried before use. R_f values refer to TLC on 0.25 mm silica gel plates (Merck F_{254}) with the same eluent used for the chromatographic purifications. Melting points (mp) were determined on an RCH Kofler apparatus. ^{1}H and ^{13}C NMR spectra were recorded at 200 MHz and 50.3 MHz, respectively, with a Varian Gemini spectrometer, or at 400 MHz and 100 MHz, respectively, with a Varian MERCURY plus spectrometer (where specified in brackets) in $CDCl_3$ solution, unless otherwise stated: the chemical shifts for ^{1}H and ^{13}C NMR spectra are given in ppm from TMS and the coupling constants values are in Hertz. IR spectra were recorded with a Perkin-Elmer 881 spectrophotometer (cm^{-1} units). Mass spectra (EI, 70 eV) were recorded with a QMD 1000 Carlo Erba instrument by direct inlet. For some hydroxylamines, CI conditions were used (methane as reacting gas). Optical rotation measurements were carried out with a Jasco DIP-370 polarimeter. Elemental analyses were carried out with a Perkin-Elmer 2400 instrument.

3-{(Acetyloxy)[(1*R*)-1-phenyl-3-butenyl]amino}-1,2,3-trideoxy-4,5-*O*-(1-methylethylidene)-D-*ribo*-hex-1-enitol (8)

Acetic anhydride (280 µL, 2.9 mmol) was added to a stirred solution of hydroxylamine **5** (330 mg, 0.99 mmol) in THF (20 mL) and the mixture was stirred at room temperature for 2.5 h. A saturated aqueous solution of sodium carbonate (15 mL) was then added. The product was extracted with AcOEt (3x10 mL). The organic phase was dried over anhydrous Na_2SO_4, then filtered and concentrated. After purification by flash column chromatography the pure desired product **8** (338 mg, 0.90 mmol) was recovered as a white oil, 91% yield (eluent AcOEt:petroleum ether=1:3, R_f=0.31); ^{1}H NMR δ 7.40-7.35 (m, 5H), 5.95 (dt, 1H, *J* = 16.8, 10.2 Hz), 5.55 (dd, 1H, *J* = 10.2, 1.5

Hz), 5.30 (ddt, 1H, *J* = 16.8, 10.2, 6.6 Hz), 5.08 (dd, 1H, *J* = 16.8, 1.5 Hz), 4.86 (bd, 1H, *J* = 16.8 Hz), 4.81 (bd, 1H, *J* = 6.6 Hz), 4.54 (dd, 1H, *J* = 11.0, 2.2 Hz), 4.33 (dt, 1H, *J* = 8.8, 5.1 Hz), 4.10 (dd, 1H, *J* = 8.8, 5.9 Hz), 4.01 (dd, 1H, *J* = 9.5, 5.9 Hz), 3.54 (td, 1H, *J* = 11.0, 5.1 Hz), 3.35 (bt, 2H, *J* = 9.5 Hz), 2.55-2.45 (m, 1H), 2.16 (s, 3H), 1.24 (s, 3H), 1.19 (s, 3H); ^{13}C NMR δ 170.9 (s, 1C), 137.1 (t, 1C), 133.9 (d, 1C), 130.8 (d, 1C), 129.0 (d, 2C), 128.6 (d, 2C),128.5 (d, 1C), 122.8 (t, 1C), 117.0 (t, 1C), 108.1 (s, 1C), 78.2 (d, 1C), 75.4 (d, 1C), 69.3 (d, 1C), 65.6 (d, 1C), 59.5 (t, 1C), 38.5 (t, 1C), 27.3 (q, 1C), 25.3 (q, 1C), 19.2 (q, 1C); IR (KBr) 3438 (m), 3082 (w), 3030 (w), 2987 (m), 2935 (m), 2892 (w), 1758 (s), 1456 (m), 1382 (m), 1370 (m), 1217 (s), 1065 (s) cm^{-1}; MS m/z (rel. int.) 376 (M$^+$, 1), 360 (3), 334 (12), 276 (13), 244 (54), 234 (18), 164 (16), 144 (13), 131 (100), 114 (90), 104 (56), 91 (62), 77 (19), 59 (81); Anal. calcd. for $C_{21}H_{29}NO_5$: C, 67.18; H, 7.79; N, 3.73 Found: C, 67.17; H, 8.12; N, 3.71; $[\alpha]_D^{20}$ = -85 (c = 0.78, CH_2Cl_2).

3-{(Acetyloxy)[(1*S*)-1-phenyl-3-butenyl]amino}-1,2,3-trideoxy-4,5-*O*-(1-methylethylidene)-D-*ribo*-hex-1-enitol (9)

Acetic anhydride (280 μL, 2.9 mmol) was added to a stirred solution of hydroxylamine **6** (329 mg, 0.99 mmol) in THF (20 mL) and the mixture was stirred at room temperature for 4 h. A saturated aqueous solution of sodium carbonate (15 mL) was then added. The product was extracted with AcOEt (3x10 mL). The organic phase was dried over anhydrous Na_2SO_4, then filtered and concentrated. After purification by flash column chromatography the pure desired product **9** (282 mg, 0.75 mmol) was recovered as a colorless oil, 76% yield (eluent AcOEt:petroleum ether=1:4, R_f=0.34); ^{1}H NMR δ 7.36-7.23 (m, 5H), 6.19 (dt, 1H, *J* = 16.8, 9.5 Hz), 6.09-5.93 (m, 1H), 5.33 (d, 1H, *J* = 16.8 Hz), 5.25 (dd, 1H, *J* = 10.2, 1.1 Hz), 5.17 (dd, 1H, *J* = 13.9, 1.5 Hz), 5.44 (dd, 1H, *J* = 9.5, 1.1 Hz), 4.81 (d, 1H, *J* = 9.5 Hz), 4.65 (bd, 1H, *J* = 9.5 Hz), 4.46 (dt, 1H, *J* = 9.5, 5.1 Hz), 4.05-3.78 (m, 2H), 3.63 (dt, 1H, *J* = 10.2, 5.1 Hz), 2.80-2.50 (m, 2H), 1.55 (s, 3H), 1.41 (s, 3H), 1.33 (s, 3H); ^{13}C NMR δ 170.0 (s, 1C), 138.9 (t, 1C), 137.1 (d, 1C), 135.5 (d, 1C), 128.2 (d, 2C), 128.1 (d, 2C),127.7 (d, 1C), 119.9 (t, 1C), 115.8 (t, 1C), 107.3 (s, 1C), 78.0 (d, 1C), 76.6 (d, 1C), 73.6 (d, 1C), 60.6 (d, 1C), 60.4 (t, 1C), 30.2 (t, 1C), 27.7 (q, 1C), 25.0 (q, 1C), 18.4 (q, 1C); IR (CH_2Cl_2) 3426 (s), 3079 (w), 2991 (m), 2939 (m), 2884 (m), 1759 (s), 1638 (m), 1495 (m), 1456 (m), 1382 (s), 1370 (s), 1213 (s), 1064 (s) cm^{-1}; MS m/z (rel. int.) 375 (M$^+$, 0.1), 361 (1), 334 (2), 244 (43), 131 (4), 118 (25), 117 (100), 91 (10), 77 (2), 59 (14); Anal. calcd. for $C_{21}H_{29}NO_5$: C, 67.18; H, 7.78; N, 3.73 Found: C, 67.03; H, 8.09; N, 3.66; $[\alpha]_D^{25}$ = +62 (c = 0.96, CH_2Cl_2).

1,2,3,4-Tetradeoxy-4-{acetyl[(1*S*)-1-phenylbut-3-enyl]amino}-5,6-*O*-(1-methylethylidene)-D-ribo-hept-1-enitol (10)

Acetic anhydride (390 μL, 4.1 mmol) was added to a stirred solution of hydroxylamine **7** (478 mg, 1.4 mmol) in THF (14 mL) and the mixture was heated under reflux for 1.5 h. After cooling, a saturated aqueous solution of sodium carbonate (20 mL) was added. The product was extracted with ethyl acetate (3x12 mL). The organic phase was dried over anhydrous Na_2SO_4, then filtered and concentrated. After purification by flash column chromatography the pure desired product **10** (506 mg, 1.3 mmol) was recovered as an oil, 93% yield (eluent ethyl acetate:petroleum ether=1:5, R_f=0.25); ^{1}H NMR δ 7.28-7.21 (m, 5H), 6.02 (ddt, 1H, *J* = 16.8, 10.3, 6.6 Hz), 5.35 (ddt, 1H, *J* = 16.8, 10.3, 6.6 Hz), 5.12 (dd, 1H, *J* = 16.8, 2.1 Hz), 5.05 (dd, 1H, *J* = 10.3, 2.1 Hz), 4.96 (dd, 1H, *J* = 16.8, 2.1 Hz), 4.89 (dd, 1H, *J* = 10.3, 2.1 Hz), 4.58 (bm, 1H), 4.42 (dt, 1H, *J* = 8.8, 5.2 Hz), 4.24 (dd, 1H, *J* = 11.0, 5.2 Hz), 4.00 (dd, 1H, *J* = 9.5, 5.9 Hz), 3.89 (bd, 1H, *J* = 11.0 Hz), 3.62 (m, 1H), 2.80-2.41 (m, 4H), 1.60 (s, 3H), 1.43 (s, 3H), 1.33 (s, 3H); ^{13}C NMR δ 170.1 (s, 1C), 138.5 (s, 1C), 137.1 (d, 1C), 133.5 (d, 1C), 129.0 (d, 2C), 128.0 (d, 2C), 127.9 (d, 1C), 117.7 (t, 1C), 116.2 (t, 1C), 107.4 (s, 1C), 78.1 (d, 1C), 76.7 (d, 1C), 69.7 (d, 1C), 61.0 (d, 1C), 60.5 (t, 1C), 36.9 (t, 1C), 30.4 (t, 1C), 27.8 (q, 1C), 25.1 (q, 1C), 18.4 (q, 1C); IR ($CDCl_3$) 3424 (m), 3079 (w), 3033 (w), 2988 (m), 2937 (m), 2891 (w), 1759 (s), 1639 (m), 1456 (m), 1370 (m), 1213 (s), 1064(s) cm^{-1}; MS (CI) m/z (rel. int.) 404 (M^++15, 17), 389 (M^+, 100), 338 (9), 324 (15), 246 (21), 233 (22), 213 (26), 200 (34), 65 (13), 51 (10); Anal. calcd. for $C_{22}H_{31}NO_5$: C, 67.84; H, 8.02; N, 3.60 Found: C, 67.71; H, 8.22; N, 3.82; $[\alpha]_D^{24}$ = +18 (c = 1.00, CH_2Cl_2).

{(4*R*,5*S*)-5-[(2*S*,6*R*)-1-(Acetyloxy)-6-phenyl-1,2,5,6-tetrahydro-2-pyridinyl]-2,2-dimethyl-1,3-dioxolan-4-yl}methanol (12)

O-protected hydroxylamine **8** (373 mg, 0.99 mmol) was dissolved in CH_2Cl_2 (15 mL) and 5% of Grubbs' Catalyst 2nd Generation (42 mg, 0.042 mmol) was added. The mixture was heated under reflux and after 1.5 h it was cooled and concentrated. It was impossible to purify the product by flash column chromatography because it was not stable on silica gel. Crude product **12** was obtained in quantitative yield as a brown oil. Proton spectrum showed some broad signals (probably due to slowly equilibrating conformers); ^{1}H NMR δ 7.37-7.22 (m, 5H), 5.91-5.64 (m, 2H), 4.15-3.73 (m, 6H), 2.45-2.22 (m, 2H), 1.51 (bs, 3H), 1.40 (s, 3H), 1.29 (s, 3H).

{(4*R*,5*S*)-5-[(2*S*,6*S*)-1-(Acetyloxy)-6-phenyl-1,2,3,6-tetrahydro-2-pyridinyl]-2,2-dimethyl-1,3-dioxolan-4-yl}methanol (13)

O-protected hydroxylamine **9** (248 mg, 0.66 mmol) was dissolved in CH_2Cl_2 (13 mL) and 5% of Grubbs' Catalyst 2nd Generation (28 mg, 0.033 mmol) was added. The mixture was heated under reflux and after 2 h it was cooled and concentrated. It was impossible to purify the product by flash

column chromatography because it was not stable on silica gel. Crude product **13** was obtained in quantitative yield as a brown oil. Proton spectrum showed some broad signals (probably due to slowly equilibrating conformers); ^{1}H NMR δ 7.56-7.46 (m, 2H), 7.44-7.31 (m, 3H), 6.31-6.21 (m, 1H), 5.85-5.75 (m, 1H), 4.73 (bs, 1H), 4.33 (dd, 1H, *J*.= 11.7, 3.7 Hz), 4.20 (dt, 1H, *J*.= 10.3, 5.0 Hz), 4.00 (dd, 1H, *J*.= 10.3, 5.1 Hz), 3.33-3.16 (m, 2H), 2.59-2.23 (m, 2H), 2.11 (s, 3H), 1.29 (s, 3H), 1.26 (s, 3H).

{(4*R*,5*S*)-5-[(2*S*,7*S*)-1-(Acetyloxy)-7-phenyl-2,3,6,7-tetrahydro-1*H*-azepin-2-yl]-2,2-dimethyl-1,3-dioxolan-4-yl}methanol (14)

O-Protected hydroxylamine **10** (501 mg, 1.30 mmol) was dissolved in CH_2Cl_2 (20 mL) and 5% of Grubbs' Catalyst 2nd Generation (55 mg, 0.065 mmol) was added. The mixture was heated under reflux and after 5.5 h it was cooled and concentrated. The crude was purified by flash column chromatography (eluent ethyl acetate:petroleum benzine=1:3, R_f=0.18) to get the pure product **14** (460 mg, 1.27 mmol) as an oil, 98% yield. Proton spectrum showed some broad signals (probably due to slowly equilibrating conformers) which could not be resolved even recording spectra at different temperatures; ^{1}H NMR δ 7.31-7.25 (m, 5H), 5.86-5.81 (m, 2H), 4.35-4.22 (m, 2H), 4.17-4.01 (bm, 1H), 3.95-3.81 (m, 2H), 3.79-3.64 (bm, 1H), 3.05-2.83 (bm, 1H), 2.76-2.47 (m, 2H), 2.35 (dd, 1H, *J* = 17.1, 6.3 Hz), 1.83 (s, 3H), 1.38 (s, 3H), 1.32 (s, 3H); ^{13}C NMR δ 168.9 (s, 1C), 140.9 (s, 1C), 130.8 (d, 1C), 129.3 (d, 1C), 128.2 (d, 2C), 127.7 (d, 2C), 127.6 (d, 1C), 107.5 (s, 1C), 78.3 (d, 1C), 75.6 (d, 1C), 70.6 (d, 1C), 62.2 (d, 1C), 60.7 (t, 1C), 32.2 (t, 1C), 27.8 (q, 1C), 25.3 (q, 1C), 24.8 (t, 1C), 18.8 (q, 1C); IR ($CDCl_3$) 3423 (m), 3031 (w), 3008 (w), 2985 (w), 2930 (w), 2896 (w), 1759 (s),1660 (w),1451 (w), 1368 (m), 1228 (s), 1200 (m), 1060 (m), 1038 (m) cm^{-1}; MS (CI) m/z (rel. int.) 361 (M^+, 22), 131 (11), 85 (30), 71 (60), 57 (100), 55 (74); Anal. calcd. for $C_{20}H_{27}NO_5$: C, 66.46; H, 7.53; N, 3.88 Found: C, 66.43; H, 7.58; N, 3.78; $[\alpha]_D^{25}$ = +38 (c = 1.1, $CHCl_3$).

{(4*R*,5*S*)-2,2-Dimethyl-5-[(2*S*,6*R*)-6-phenyl-1,2,5,6-tetrahydro-2-pyridinyl]-1,3-dioxolan-4-yl}methanol (15)

The crude **12** (345 mg, 0.99 mmol) was dissolved in MeOH saturated with $KHCO_3$ (10 mL). The solution was stirred at room temperature for 24 h, and then the mixture was concentrated, H_2O (10 mL) was added and the product was extracted with AcOEt (3x10 mL). The organic phase was dried over anhydrous Na_2SO_4, then filtered and concentrated. It was impossible to purify the product by flash column chromatography because it was not stable on silica gel. Crude hydroxylamine was obtained in quantitative yield; ^{1}H NMR δ 7.46-7.20 (m, 5H), 5.99-5.86 (m, 1H), 5.55 (bd, 1H *J* = 9.5 Hz), 4.50-4.41 (m, 1H), 4.22-3.97 (m, 2H), 3.90-3.67 (m, 2H), 3.61-3.43 (m, 1H), 2.54-2.16 (m,

2H), 1.37 (s, 3H), 1.26 (s, 1H). The crude hydroxylamine (303 mg, 0.99 mmol) was then dissolved in 90% AcOH (25 mL). Zinc powder (260 mg, 3.97 mmol) was added and the mixture was stirred at room temperature for 1 h. Then the acid was removed and a saturated aqueous solution of $NaHCO_3$ (20 mL) was carefully added. The product was extracted with AcOEt (3x20 mL). The organic phase was dried over anhydrous Na_2SO_4, then filtered and concentrated. The crude reaction mixture was purified by flash column chromatography to afford product **15** in 64% yield (calculated on 50% conversion of unprotected hydroxylamine) from **8**; ^{1}H NMR δ 7.47-7.26 (m, 5H), 6.06-6.94 (m, 1H), 5.72 (bd, 1H, *J* = 10.9 Hz), 4.36-4.30 (m, 1H), 4.25-4.15 (m, 2H), 3.93 (dd, 1H, *J* = 10.3, 4.4 Hz), 3.81-3.72 (m, 2H), 2.56-2.19 (m, 2H), 1.47 (s, 3H), 1.36 (s, 1H).

{(4*R*,5*S*)-2,2-Dimethyl-5-[(2*S*,6*S*)-6-phenyl-1,2,3,6-tetrahydro-2-pyridinyl]-1,3-dioxolan-4-yl}methanol (16)

214 mg of crude **13** (0.62 mmol) were dissolved in MeOH saturated with $KHCO_3$ (20 mL). The solution was stirred at room temperature for 24 h, and then the mixture was concentrated, H_2O (15 mL) was added and the product was extracted with AcOEt (3x15 mL). The organic phase was dried over anhydrous Na_2SO_4, then filtered and concentrated. It was impossible to purify the product by flash column chromatography because it was not stable on silica gel. Crude hydroxylamine was obtained in quantitative yield; ^{1}H NMR δ 8.20-8.05 (bs, 1H), 7.50-7.30 (m, 5H), 6.32-5.23 (m, 1H), 5.81 (bd, 1H *J* = 10.0 Hz), 4.72 (bs, 1H), 4.38-4,31 (m, 1H), 4.28-4.17 (m, 1H), 3.33 (dd, 1H, *J* = 10.0, 4.7 Hz), 3.20-3.07 (m, 1H), 2.57-2.39 (m, 2H), 2.27-2.10 (m, 1H), 1.32 (s, 3H), 1.30 (s, 1H). The crude hydroxylamine (189 mg, 0.62 mmol) was then dissolved in 90% AcOH (18 mL). Zinc powder (162 mg, 2.48 mmol) was added and the mixture was stirred at room temperature for 1.5 h. Then the acid was removed and a saturated aqueous solution of $NaHCO_3$ (15 mL) was carefully added. The product was extracted with AcOEt (3x15 mL). The organic phase was dried over anhydrous Na_2SO_4, then filtered and concentrated. It was impossible to purify the product by flash column chromatography because it was not stable on silica gel. Crude product **16** was obtained in 77% yield from **9**; ^{1}H NMR δ 7.42-7.30 (m, 5H), 7.11-6.85 (bs, 3H), 6.91-6.81 (m, 1H), 4.73 (bs, 1H), 4.32 (dt, 1H, *J*.= 10.2, 5.1 Hz), 4.18 (dd, 1H, *J*.= 9.5, 5.9 Hz), 3.48 (m, 1H, *J*.= 11.7, 4.4 Hz),3.24-3.12 (m, 1H), 2.86 (bt, 1H, *J*.= 10.2) 2.53-2.12 (m, 2H), 1.31 (s, 3H), 1.28 (s, 3H).

{(4*R*,5*S*)-2,2-Dimethyl-5-[(2*S*,7*S*)-7-phenyl-2,3,6,7-tetrahydro-1*H*-azepin-2-yl]-1,3-dioxolan-4-yl}methanol (17)

N-protected hydroxylamine **14** (168 mg, 0.46 mmol) was dissolved in MeOH saturated with $KHCO_3$ (20 mL). The solution was stirred at room temperature. After 12 h the mixture was

concentrated, H_2O (10 mL) was added and the product was extracted with ethyl acetate (3x10 mL). The organic phase was dried over anhydrous Na_2SO_4, then filtered and concentrated. The crude was obtained as an analytically pure white solid in 100% yield (eluent ethyl acetate:petroleum ether=1:2, R_f=0.47); mp = 170-172 °C; ^{1}H NMR (400 MHz) δ 7.37-7.29 (m, 5H), 6.27 (bs, 1H, exchanging proton), 5.87 (bm, 2H), 5.78 (bm, 1H, exchanging proton), 4.34 (bs, 1H), 4.17 (bs, 1H), 3.97 (bs, 1H), 3.74 (bs, 1H), 3.09 (bd, 1H), 3.01-2.75 (m, 2H), 2.48-2.28 (m, 1H), 2.21 (bm, 1H), 2.15 (bdd, 1H), 1.37 (s, 3H), 1.33 (s, 1H); ^{13}C NMR δ 142.5 (s, 1C), 130.3 (bd, 2C), 128.4 (d, 2C), 127.6 (d, 3C), 107.1 (s, 1C), 77.1 (d, 2C), 72.2 (bd, 1C), 63.3 (bd, 1C), 60.7 (t, 1C), 32.2 (bt, 1C), 28.1 (q, 1C), 25.4 (q, 1C), 24.9 (bt, 1C); IR (KBr) 3270 (m), 3064 (w), 3027 (w), 2990 (w), 2934 (w), 2897 (w), 1651 (w), 1456 (m), 1441 (m), 1379 (m), 1369 (m), 1242 (m), 1223 (m), 1076 (m), 1056 (m), 1036 (s), 875 (m), 752 (m) 699 (m) cm^{-1}; MS (CI) m/z (rel. int.) 319 (M^+, 27), 111 (21), 85 (41), 71 (90), 69 (100), 59 (90), 57 (82), 55 (69); Anal. calcd. for $C_{18}H_{25}NO_4$: C, 67.69; H, 7.89; N, 4.39 Found: C, 67.42; H, 7.83; N, 4.50; $[\alpha]_D^{22}$ = +76 (c = 0.44, $CHCl_3$). The crude hydroxylamine (147 mg, 0.46 mmol) was then dissolved in 90% AcOH (8 mL). Zinc powder (150 mg, 2.3 mmol) was added and the mixture was stirred at rt for 2 h. Then the acid was removed and a saturated aqueous solution of sodium bicarbonate (15 mL) was carefully added. The product was extracted with ethyl acetate (3x15 mL). The organic phase was dried over anhydrous Na_2SO_4, then filtered and concentrated. The crude reaction mixture was purified by flash column chromatography to afford product **17** (126 mg, 0.41 mmol) as a white hygroscopic solid, 90% yield (eluent ethyl acetate:petroleum benzine=1:2, R_f=0.30); mp = 139-140 °C; ^{1}H NMR (400 MHz) δ 7.37-7.22 (m, 5H), 5.89-5.73 (m, 2H), 4.39 (d, 1H, *J* = 10.6 Hz), 4.26 (ddd, 1H, *J* = 9.4, 5.5, 3.3 Hz), 4.12 (dd, 1H, *J* = 9.2, 5.5 Hz), 3.81 (dd, 1H, *J* = 11.4, 9.4 Hz), 3.69 (td, 1H, *J* = 9.2, 2.6 Hz), 3.55 (dd, 1H, *J* = 11.4, 3.3 Hz), 2.83-2.71 (m, 1H), 2.61 (dd, 1H, *J* = 17.2, 6.6 Hz), 2.56-2.44 (m, 1H), 2.37 (dd, 1H, *J* = 17.2, 6.6 Hz), 1.39 (s, 3H), 1.33 (s, 1H); ^{13}C NMR δ 138.8 (s, 1C), 129.0 (d, 1C), 128.9 (d, 1C), 128.8 (d, 2C), 128.7 (d, 1C), 126.9 (d, 2C), 108.4 (s, 1C), 76.6 (d, 1C), 76.2 (d, 1C), 60.8 (t, 1C), 59.9 (d, 1C), 55.0 (d, 1C), 33.4 (t, 1C), 29.0 (t, 1C), 28.0 (q, 1C), 25.5 (q, 1C); IR (conc. $CHCl_3$ solution) 3250-3100 (m), 3026 (w), 2988 (w), 2934 (m), 2861 (w), 1653 (w), 1602 (w), 1494 (m), 1454 (m), 1434 (m), 1381 (s), 1372 (m), 1220 (s), 1044 (s); IR (dil. $CHCl_3$ solution) 3452 (w), 3230-3180 (w), 3053 (m), 3029 (w), 2988 (w), 2935 (w), 2856 (w), 1649 (w), 1493 (w), 1455 (w), 1435 (w), 1382 (m), 1372 (m), 1220 (m), 1040 (m) cm^{-1}; MS (CI) m/z (rel. int.) 303 (M^+, 100), 285 (91), 276 (22), 219 (38), 109 (23), 71 (42), 57 (70), 55 (45); Anal. calcd. for $C_{18}H_{25}NO_3 \cdot 1/3\ H_2O$: C, 69.87; H, 8.36; N, 4.53 Found: C, 70.06; H, 8.49; N, 4.51; $[\alpha]_D^{21}$ = +60 (c = 1, $CHCl_3$).

(3a*R*,6*R*,9a*S*,9b*S*)-2,2-Dimethyl-6-phenyl-3a,4,6,7,9a,9b-hexahydro[1,3]dioxolo[4,5-*a*]indolizine (18)

Amine **15** (150 mg, 0.26 mmol) was dissolved in dry pyridine (4.0 mL). Trific anhydride (0.13 mL, 0.79 mmol) was then added and the mixture was stirred at room temperature for 2.5 h. Then AcOEt (20 mL) was added and washed with water (2x15 mL). The organic phase was dried over anhydrous Na_2SO_4, then filtered and concentrated. The crude was purified by flash column chromatography to get product **18** (40 mg, 0.15mmol) as a yellow solid, 58% yield (eluent AcOEt:ether=1:8, R_f=0.33); m.p. = 87-88 °C; ^{1}H NMR (400 MHz) δ 7.42-7.39 (m, 2H), 7.35-7.31 (m, 2H), 7.27-7.23 (m, 1H), 5.90-5.86 (m, 1H), 5.79 (bd, 1H, *J* = 10.0 Hz), 4.67-4.63 (m, 1H), 4.41-4.39 (m, 1H), 3.97 (bdd, 1H, *J* = 10.4 Hz), 3.53 (bs, 1H), 2.82 (bdd, 1H, *J* = 10.0, 5.6 Hz), 2.44-2.38 (m, 2H), 2.33-2.28 (m, 1H), 1.56 (s, 3H), 1.33 (s, 3H); ^{13}C NMR (100 MHz) δ 128.2 (d, 2C), 127.1 (d, 2C), 127.0 (d, 1C), 126.8 (d, 1C), 125.9 (d, 1C), 115.5 (s, 1C), 112.5 (s, 1C), 83.0 (d, 1C), 78.3 (d, 1C), 67.3 (d, 1C), 59.5 (d, 1C), 52.5 (t, 1C), 28.6 (t, 1C), 27.0 (q, 1C), 25.5 (q, 1C); IR (CH_2Cl_2) 3685 (m), 3599 (w), 3030 (m) 2936 (m), 2832 (w), 1710 (w), 1602 (m), 1495 (w), 1452 (w), 1382 (s), 1370 (s), 1285 (m), 1209 (s), 1119 (m), 1056 (s) cm^{-1}; MS m/z (rel. int.) 271 (M^+, 67), 213 (24), 212 (74), 170 (100), 156 (30), 128 (19), 104 (13), 91 (35), 67 (31); Anal. calcd. for $C_{17}H_{21}NO_2$: C, 75.25; H, 7.80; N, 5.16 Found: C, 75.18; H, 7.99; N, 5.31; $[\alpha]_D^{24}$ = +3 (c = 0.30, CH_2Cl_2).

1D NOESY experiments on **18** were carried out with a Varian MERCURY plus spectrometer (H-400 MHz) using the automatic pulse sequence NOESY 1D (main parameters used: sw=3998, at=2.664, ss=-2, d1=1, nt=64, spin off; lb=0.38 for the processing). The sample (10 mM in $CDCl_3$) was carefully degassed by bubbling argon through the solution and the experiments were run at 25 °C varying the mixing times (150 ms, 350 ms, 500 ms, 800 ms and 1000 ms).

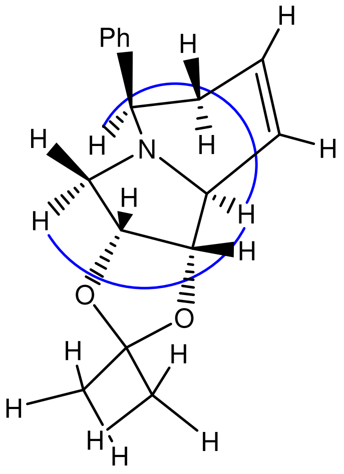

(3a*R*,6*S*,9a*S*,9b*S*)-2,2-Dimethyl-6-phenyl-3a,4,6,9,9a,9b-hexahydro[1,3]dioxolo[4,5-*a*]indolizine (19)

Amine **16** (138 mg, 0.48 mmol) was dissolved in dry pyridine (5.0 mL). Trific anhydride (0.12 mL, 0.78 mmol) was then added and the mixture was stirred at room temperature for 2 h. Then AcOEt (20 mL) was added and washed with water (2x15 mL). The organic phase was dried over anhydrous Na_2SO_4, then filtered and concentrated. The crude was purified by flash column chromatography to get product **19** (101 mg, 0.46 mmol) as a yellow solid, 97% yield (eluent AcOEt:petroleum ether=1:3, R_f=0.30); m.p. = 54-55 °C; ^{1}H NMR (400 MHz) δ 7.36-7.33 (m, 2H), 7.29-7.26 (m, 3H), 6.01-5.96 (m, 1H), 5.87-5.83 (m, 1H), 4.74-4.70 (m, 1H), 4.40 (bs, 1H), 4.30 (dd, 1H, *J* = 6.8, 4.0 Hz), 3.19 (dd, 1H, *J* = 9.6, 6.0 Hz), 2.92-2.87 (m, 1H), 2.41 (bdt, 1H, *J* = 17.6, 4.8 Hz), 2.29 (dd, 1H, *J* = 9.6, 4.4 Hz), 2.16-2.09 (m, 1H), 1.36 (s, 3H), 1.29 (s, 3H); ^{13}C NMR (100 MHz) δ 138.1 (s, 2C), 128.8 (d, 2C), 127.9 (d, 2C), 127.4 (d, 1C), 127.2 (d, 1C), 124.6 (d, 1C), 112.9 (s, 1C), 85.3 (d, 1C), 77.5 (d, 1C), 59.9 (d, 1C), 56.5 (d, 1C), 55.9 (t, 1C), 28.1 (t, 1C), 27.0 (q, 1C), 25.4 (q, 1C); IR (CH_2Cl_2) 3686 (m), 3600 (w), 3033 (m) 2985 (m), 2934 (s), 2835 (m), 1730 (w), 1604 (m), 1491 (m), 1452 (m), 1382 (s), 1374 (s), 1277 (m), 1208 (s), 1079 (m), 1055 (s) cm^{-1}; MS m/z (rel. int.) 271 (M^+, 100), 213 (19), 212 (46), 194 (28), 170 (37), 156 (14), 130 (44), 115 (11), 91 (9), 80 (22), 71 (13), 69 (25), 57 (50); Anal. calcd. for $C_{17}H_{21}NO_2$: C, 75.25; H, 7.80; N, 5.16 Found: C, 75.07; H, 7.97; N, 5.05; $[\alpha]_D^{24}$ = -190 (c = 0.94, $CHCl_3$).

1D NOESY experiments on **19** were carried out with a Varian MERCURY plus spectrometer (H-400 MHz) using the automatic pulse sequence NOESY 1D (main parameters used: sw=3998, at=2.664, ss=-2, d1=1, nt=64, spin off; lb=0.38 for the processing). The sample (10 mM in $CDCl_3$) was carefully degassed by bubbling argon through the solution and the experiments were run at 25 °C varying the mixing times (150 ms, 350 ms, 500 ms, 800 ms and 1000 ms).

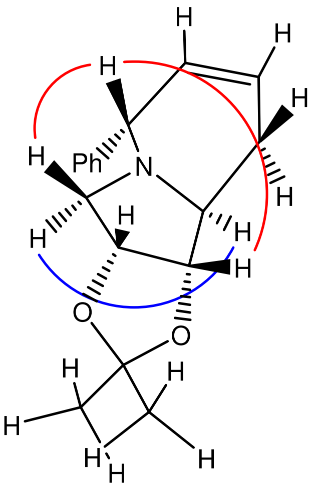

(3a*R*,6*S*,10a*S*,10b*S*)-2,2-Dimethyl-6-phenyl-3a,6,7,10,10a,10b-hexahydro-4*H*-[1,3]dioxolo[3,4]pyrrolo[1,2-*a*]azepine (20)

Amine **17** (21 mg, 0.07 mmol) was dissolved in dry pyridine (0.5 mL). Trific anhydride (17 µL, 0.1 mmol) was then added and the mixture was stirred at rt for 2 h. Then ethyl acetate (10 mL) was added and washed with water (2x10 mL). The organic phase was dried over anhydrous Na_2SO_4, then filtered and concentrated. The crude was purified by flash column chromatography to get product **20** (18 mg, 0.06 mmol) as a white solid, 90% yield (eluent ethyl acetate:petroleum ether=1:20, R_f=0.48); mp = 79-81 °C; ^{1}H NMR (400 MHz) δ 7.42-7.40 (m, 2H), 7.30-7.26 (m, 2H), 7.22-7.18 (m, 1H), 5.77-5.67 (m, 2H), 4.65 (dd, 1H, *J* = 6.4, 4.9 Hz), 4.34 (d, 1H, *J* = 6.4 Hz), 3.97 (d, 1H, *J* = 11.7 Hz), 3.70 (d, 1H, *J* = 11.1 Hz), 2.85 (d, 1H, *J* = 11.5 Hz), 2.76 (m, 1H), 2.52-2.43 (m, 1H), 2.48 (dd, 1H, *J* = 11.5, 4.9 Hz), 2.12 (dd, 1H, *J* = 17.5, 7.5 Hz), 1.84 (dd, 1H, *J* = 17.2, 6.5 Hz), 1.60 (s, 3H), 1.32 (s, 1H); ^{13}C NMR (100 MHz) δ 145.8 (s, 1C), 130.0 (d, 1C), 129.0 (d, 1C), 128.3 (d, 2C), 126.7 (d, 3C), 111.1 (s, 1C), 86.7 (d, 1C), 79.3 (d, 1C), 66.6 (d, 1C), 66.3 (d, 1C), 57.0 (t, 1C), 37.1 (t, 1C), 26.8 (q, 1C and t, 1C), 25.4 (q, 1C); IR ($CDCl_3$) 3023 (w), 2992 (w), 2930 (m), 2793 (w), 1653 (w), 1488 (w), 1452 (m), 1380 (m), 1371 (s), 1206 (m), 1147 (s), 1060 (s) cm^{-1}; MS m/z (rel. int.) 285 (M^+, 51), 270 (16), 231 (100), 216 (82), 185 (22), 173 (28), 156 (31), 143 (28), 129 (59), 117 (50), 104 (30), 91 (41), 57 (28); Anal. calcd. for $C_{18}H_{23}NO_2$: C, 75.76; H, 8.12; N, 4.91 Found: C, 75.57; H, 8.12; N, 5.09; $[\alpha]_D^{28}$ = –13 (c = 0.72, CH_2Cl_2).

1D NOESY experiments on **20** were carried out with a Varian MERCURY plus spectrometer (H-400 MHz) using the automatic pulse sequence NOESY 1D (main parameters used: sw=3998, at=2.664, ss=-2, d1=1, nt=64, spin off; lb=0.38 for the processing). The sample (10 mM in $CDCl_3$) was carefully degassed by bubbling argon through the solution and the experiments were run at 24 °C varying the mixing times (150 ms, 350 ms, 500 ms, 800 ms and 1000 ms).

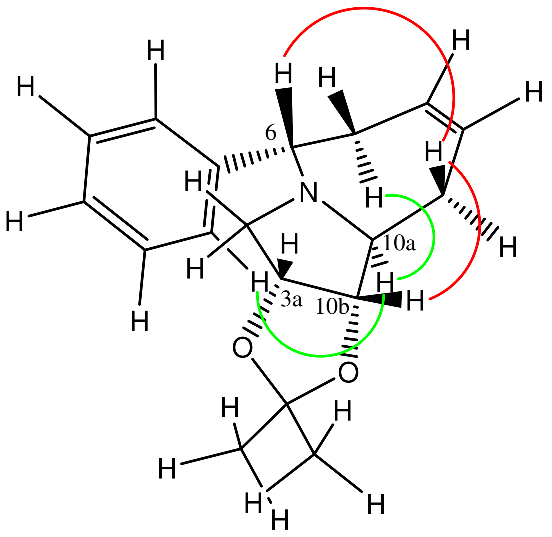

(1*S*,2*R*,5*R*,8a*S*)-1,2-Dihydroxy-5-phenyl-1,2,3,5,6,8a-hexahydroindolizinium chloride (21)
Compound **18** (21 mg, 0.08 mmol) was dissolved in an acidic solution obtained by addition of 37% HCl (50 μL) to methanol (2 mL). The mixture was stirred at room temperature for 2 h and then concentrated to give analytically pure **21** (20 mg, 0.07 mmol) in 90% yield as a very hygroscopic brown solid, which decomposed without melting over 280 °C; ^{1}H NMR (400 MHz, CD_3OD) δ 7.46 (bs, 5H), 5.70 (bt, 1H, *J* = 9.7 Hz), 5.57-5.52 (m, 1H), 4.83 (bd, 1H, *J* = 11.7 Hz), 4.09-4.05 (m, 1H), 4.02 (dd, 1H, *J* = 6.2, 4.0 Hz), 3.94 (bdd, 1H, *J* = 10.2, 6.2 Hz), 3.15 (dd, 1H, *J* = 12.5, 2.7 Hz), 3.08-2.88 (m, 2H), 2.75 (bd, 1H, *J* = 12.5 Hz), 2.43 (bdd, 1H, *J* = 17.3, 5.7 Hz), 2.35 (bdd, 1H, *J* = 15.0, 9.7 Hz); ^{13}C NMR δ 134.4 (s, 1C), 131.1 (d, 1C), 130.3 (d, 2C), 130.2 (d, 2C), 127.5 (d, 1C), 122.4 (d, 1C), 76.0 (d, 1C), 69.8 (d, 1C), 69.3 (d, 1C), 61.6 (d, 1C), 51.5 (t, 1C), 24.4 (t, 1C); MS (C. I.) m/z (rel. int.) 231 (38), 170 (100), 156 (13), 143 (15), 128 (16), 118 (19), 104 (19), 91 (68), 67 (65); Anal. calcd. for $C_{14}H_{18}ClNO_2 \cdot 2\ H_2O$: C, 55.35; H, 7.30; N, 4.61 Found: C, 55.04; H, 6.92; N, 4.36; $[\alpha]_D^{24}$ = -102 (c = 1.00, CH_3OH).

(1*S*,2*R*,5*S*,8a*S*)-1,2-Dihydroxy-5-phenyl-1,2,3,5,8,8a-hexahydroindolizinium chloride (22)
Compound **19** (11 mg, 0.04 mmol) was dissolved in an acidic solution obtained by addition of 37% HCl (50 μL) to methanol (2 mL). The mixture was stirred at room temperature for 2 h and then concentrated to give analytically pure **22** (10 mg, 0.04 mmol) in 92% yield as a highly hygroscopic brown solid, which decomposed without melting over 280 °C; ^{1}H NMR (D_2O) δ 7.49-7.40 (m, 5H); 6.32-6.15 (m, 1H); 5.95-5.78 (m, 1H); 5.15-5.02 (m, 1H); 4.27-4.12 (m, 1H); 4.00-3.85 (m, 1H); 3.78-3.58 (m, 1H); 3.47-3.30 (m, 1H); 2.90-2.64 (m, 2H); 2.53-2.27 (m, 1H); ^{13}C NMR (D_2O) δ 133.0 (d, 2C); 131.7 (d, 1C); 130.4 (d, 2C); 130.1 (d, 2C); 127.3 (d, 1C); 124.6 (d, 1C); 74.9 (d,

1C); 67.4 (d, 1C); 63.1 (d, 1C); 57.5 (d, 1C); 55.8 (t, 1C); 28.3 (t, 1C); MS m/z (rel. int.) 232 (8), 231 (52), 230 (34), 170 (55), 158 (16), 156 (16), 154 (57), 143 (22), 130 (100), 115 (43), 91 (22), 67 (11); Anal. calc. for $C_{14}H_{18}ClNO_2 \cdot 2\ H_2O$: C, 55.35; H, 7.30; N, 4.61 Found: C, 55.14; H, 6.98; N, 5.01; $[\alpha]_D^{25}$ = -152 (c = 0.32, CH_3OH).

(1*S*,2*R*,5*S*,9a*S*)-1,2-Dihydroxy-5-phenyl-2,3,5,6,9,9a-hexahydro-1*H*-pyrrolo[1,2-*a*]azepinium chloride (23)

Compound **20** (18 mg, 0.06 mmol) was dissolved in an acidic solution obtained by addition of 37% HCl (50 μL) to methanol (2 mL). The mixture was stirred at rt for 2 h and then concentrated to give analytically pure **23** (13.5 mg, 0.048 mmol) in 80% yield as a highly hygroscopic solid, which decomposed without melting over 280 °C; ^{1}H NMR (400 MHz, D_2O) δ 7.33 (m, 5H), 5.70 (bt, 1H, *J* = 9.7 Hz), 5.57-5.52 (m, 1H), 4.83 (bd, 1H, *J* = 11.7 Hz), 4.09-4.05 (m, 1H), 4.02 (dd, 1H, *J* = 6.2, 4.0 Hz), 3.94 (bdd, 1H, *J* = 10.2, 6.2 Hz), 3.15 (dd, 1H, *J* = 12.5, 2.7 Hz), 3.08-2.88 (m, 2H), 2.75 (bd, 1H, *J* = 12.5 Hz), 2.43 (bdd, 1H, *J* = 17.3, 5.7 Hz), 2.35 (bdd, 1H, *J* = 15.0, 9.7 Hz); ^{13}C NMR δ 135.7 (s, 1C), 129.8 (d, 1C), 129.5 (d, 2C), 128.1 (d, 1C), 127.6 (d, 2C), 125.7 (d, 1C), 76.3 (d, 1C), 69.8 (d, 1C), 67.8 (d, 1C), 64.6 (d, 1C), 58.0 (t, 1C), 34.8 (t, 1C), 26.3 (t, 1C) cm^{-1}; MS (CI) m/z (rel. int.) 173 (13), 123 (14), 111 (17), 85 (33), 71 (79), 69 (78), 57 (100), 55 (77); Anal. calcd. for $C_{15}H_{20}NO_2 \cdot 0.9\ H_2O$: C, 60.46; H, 7.37; N, 4.70 Found: C, 60.59; H, 7.24; N, 4.73; $[\alpha]_D^{26}$ = +24 (c = 0.97, CH_3OH).
